# Multi-organ involvement caused by *Scedosporium apiospermum* infection after near drowning: a case report and literature review

**DOI:** 10.1186/s12883-024-03637-9

**Published:** 2024-04-15

**Authors:** Yao Yao, Qian Xu, Wenwen Liang, Suqiong Ji, Mohammadreza Kosari, Shabei Xu, Zhou Zhu, Zhijuan Mao

**Affiliations:** 1 Department of Neurology, Tongji Hospital, Tongji Medical College, Huazhong University of Science and Technology, Wuhan, Hubei China; 2 Department of Hematology, Tongji Hospital, Tongji Medical College, Huazhong University of Science and Technology, Wuhan, Hubei China; 3 Second Clinical College, Tongji Hospital, Tongji Medical College, Huazhong University of Science and Technology, Wuhan, Hubei China

**Keywords:** *Scedosporium apiospermum*, Metagenomic next-generation sequencing, Normal immune function, Voriconazole, Vision loss, Multiple (brain) lesions

## Abstract

**Background:**

*Scedosporium apiospermum* (*S. apiospermum*) is a rare fungal pathogen that causes disseminated infections. It rarely affects immunocompetent individuals and has a poor prognosis.

**Case presentation:**

A 37-year-old woman presented with multiple lesions in the lungs, brain, and eyes, shortly after near drowning in a car accident. The primary symptoms were chest tightness, limb weakness, headache, and poor vision in the left eye. *S. apiospermum* infection was confirmed by metagenomic next-generation sequencing (mNGS) of intracranial abscess drainage fluid, although intracranial metastases were initially considered. After systemic treatment with voriconazole, her symptoms improved significantly; however, she lost vision in her left eye due to delayed diagnosis.

**Conclusion:**

While *S. apiospermum* infection is rare, it should be considered even in immunocompetent patients. Prompt diagnosis and treatment are essential. Voriconazole may be an effective treatment option.

**Supplementary Information:**

The online version contains supplementary material available at 10.1186/s12883-024-03637-9.

## Background

*Scedosporium apiospermum* (*S. apiospermum*), also known as “foot actinomycosis,” is a fungus commonly found in soil, sewage, and animal feces. It can cause a wide variety of infections, such as subcutaneous, pulmonary, bone, or joint infections, often resulting in severe or even fatal disseminated infections in immunocompromised hosts [1]. In addition, it can also affect immunocompetent individuals under certain circumstances, such as trauma and near-drowning incidents in contaminated water, as in our case [2]. Multi-organ involvement due to *S. apiospermum* in immunocompetent patients is a rare but serious phenomenon that can easily remain mis- or underdiagnosed.

Here we describe a young female with a normal immune system who developed multiple lesions in her lung, brain, and eyes following a car accident and near drowning in a fishpond. The pathogenic investigations confirmed *S. apiospermum*.

## Case presentation

**Fig. 1:**
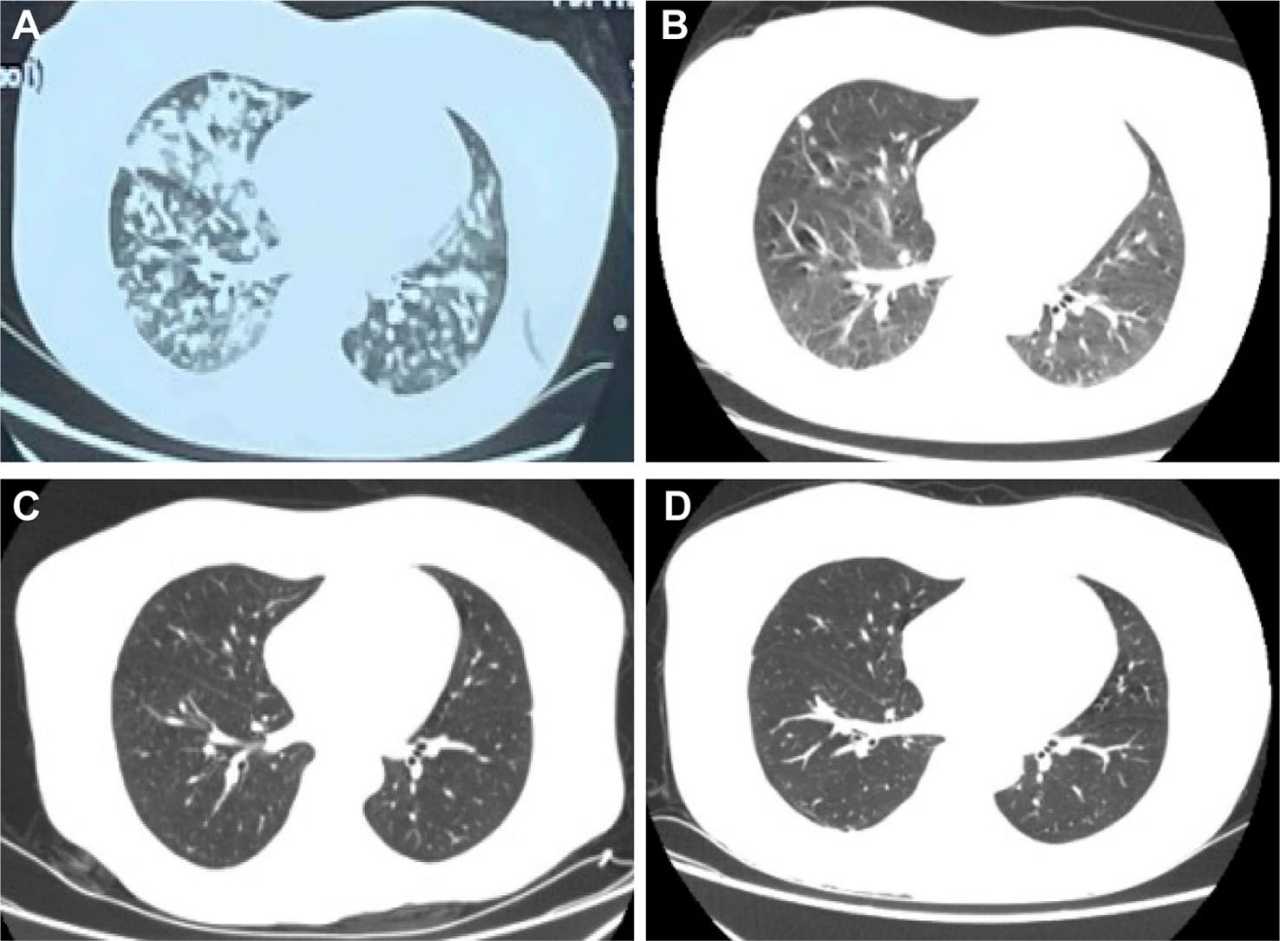
Chest CT at different stages. (**A**) In early 2023, a local lung CT scan showed multiple nodular increased density shadows in both lungs. (**B**) Five weeks later (1 week of antifungal treatment), a lung CT scan showed that the bilateral lung lesions were significantly less and smaller than before. (**C**) Nine weeks after the incident (1 month of antifungal treatment), lung CT showed that the abscess lesions were smaller than last time. (**D**) More than 8 months after the incident (more than 7 months of antifungal treatment), the bilateral lung lesions still exist

**Fig. 2:**
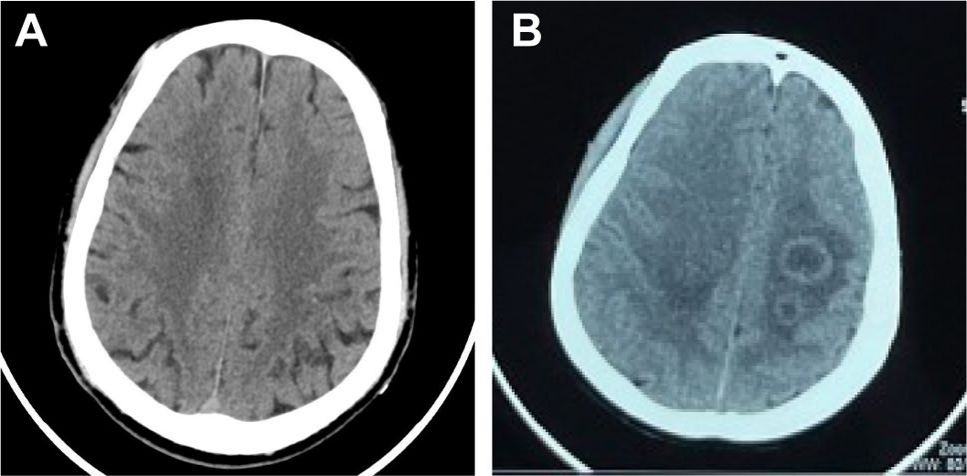
Head CT at different stages. (**A**) Head CT showed no apparent abnormalities. (**B**) Head CT scan (13 days after the accident) showed multiple rings of slightly high-density shadows with slightly low-density shadows around

**Fig. 3:**
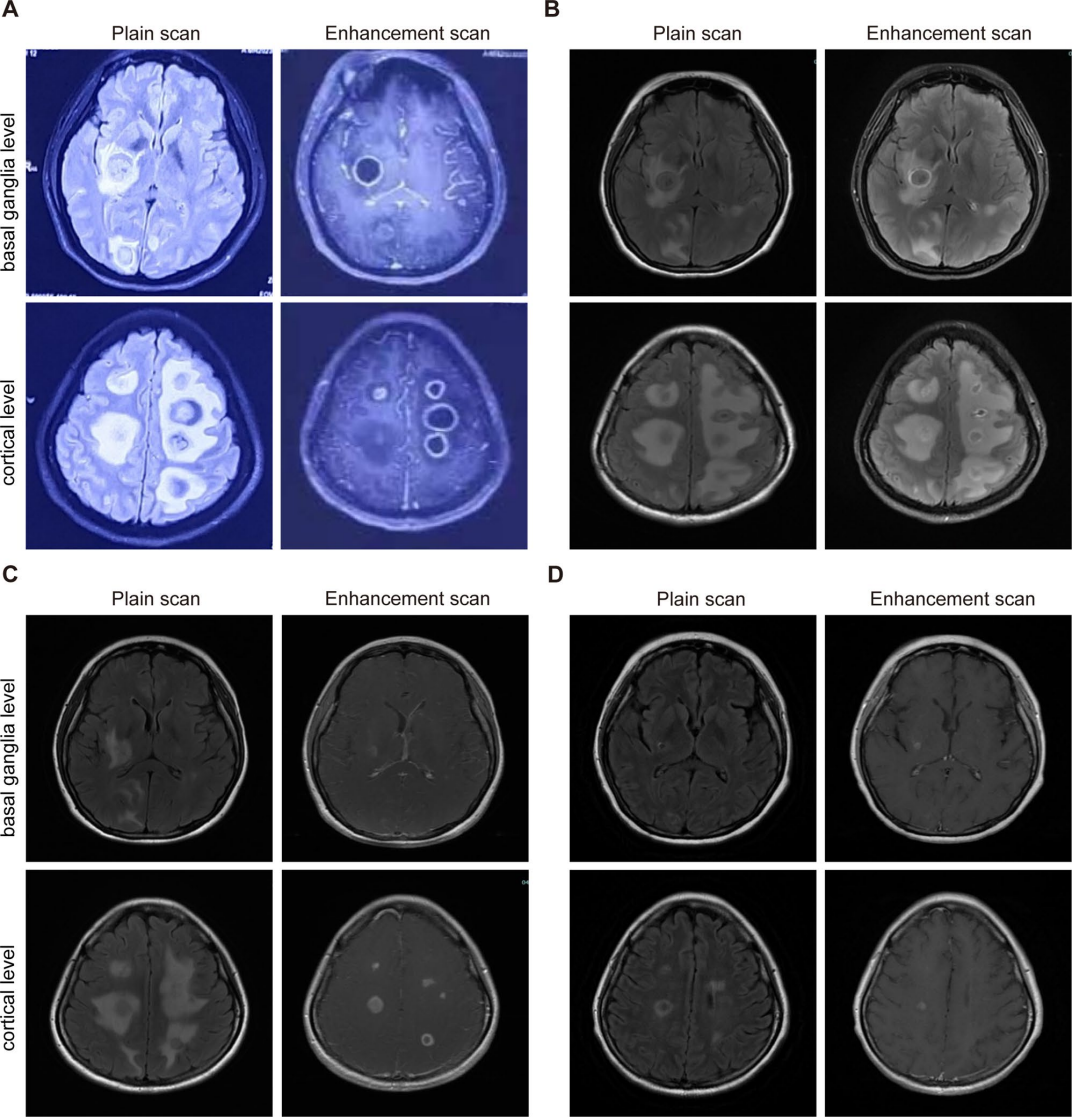
Head MRI at different stages. (**A**) Head MRI (13 days after the accident) showed multiple ring reinforcement nodules and surrounding edema in the brain. (**B**) On March 7, 2023(1 week of antifungal treatment), the head MRI showed that the brain abscess lesions were smaller than before, and the edema around the lesions was more serious. (**C**) On April 6, 2023(5 weeks of antifungal treatment), a head MRI showed that the abscess lesions were smaller and the edema was lighter than on March 7, 2023. (**D**) On October 16, 2023(more than 7 months of antifungal treatment), the brain abscess lesions still exist

A 37-year-old female patient was admitted to local hospital on January 28, 2023, due to chest tightness and dyspnea for 4 h. The patient reported no previous history of HIV/AIDS, organ transplantation, corticosteroid use, or immunosuppressive medications. Before admission, the woman had a car accident and drowned in a private fishpond. After being rescued, she developed chest tightness and breathing difficulties which were assumed to be due to water aspiration. The lung computed tomography (CT) scan (Fig. 1A) showed bilateral pulmonary edema with multiple high-density nodular shadows. The head CT showed no apparent abnormalities (Fig. 2A). She was then started on corticosteroids, diuretics, and antibiotics for 12 days which was discontinued when her respiratory symptoms were improved. However, she developed a new headache and subsequently, rapid cognitive decline, aphasia, lethargy, and impaired consciousness. Physical examination revealed significant muscle weakness in the left limb (grade 2), compared to her right limb (grade 4). The second head CT (Fig. 2B) showed multiple annular high-density shadows with slightly low-density shadows around it. A head magnetic resonance imaging (MRI) (Fig. 3A) showed multiple ring reinforcement nodules with cerebral edema, which raised the concern for multiple intracranial metastatic lesions. Thus, a positron emission tomography–computed tomography (PET/CT) was performed and revealed multiple hypermetabolic nodules within the cranium and lungs, suggesting a high probability of infection. The metagenomic next-generation sequencing (mNGS) and culture of cerebrospinal fluid (CSF) detected no specific pathogen.

After a course of antibiotics treatment, her headache aggravated, fever and vomiting occurred, and muscle strength further decreased (left limb: grade 1, right limb: grade 3). Additionally, decreased vision of the left eye and mild conjunctival congestion appeared. She was then transferred to the Department of Neurosurgery of our hospital.

**Fig. 4:**
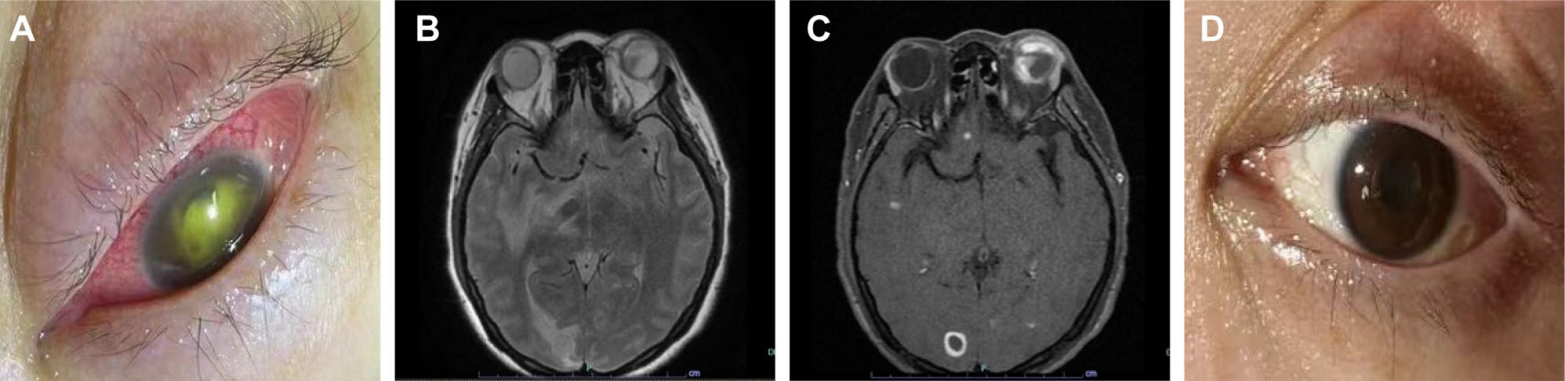
Ocular appearance and orbital MRI. (**A**) One month after the accident, left eye conjunctival congestion, anterior chamber visible numerous yellow-green exudates sealed the pupil. (**B**, **C**) On March 22, 2023 (3 weeks of antifungal treatment), an orbital MRI showed an abnormal signal of the left eyeball with enhancement. (**D**) On April 6, 2023(5 weeks of antifungal treatment), the pupil of the left eye was clear, but the eyeball atrophy and vision loss remained

The puncture drainage of intracranial lesions was performed and the pus was tested by mNGS. A total of 600ul brain abscess drainage fluid samples were collected and mixed by glass beads. DNA was extracted by column extraction method. The libraries were constructed using end repair, and specific tag sequences were introduced at the end of each library. The size of library inserts to be sequenced was determined using an Agilent 2100 Bioanalyzer (G2939BA, Agilent, USA). DNA nanospheres were prepared using a one-step DNB preparation kit (1,000,025,076, Huada Gene Technology Co. Ltd, Shenzhen, China). Sequencing was performed on the MGISEQ-200 sequencing platform. Then the reads containing sequencing adapter, low quality, short (length < 35 bp) and low complexity were removed, and aligned to the human reference genome (hg38) using STAR software (v2.7.1a) to remove the host reads. The remaining non-host reads were searched and classified against four self-built pathogenic microorganism genome databases, including bacteria, fungi, parasites and viruses. Finally, *S. apiospermum* was detected with a coverage of 99.45%. At our hospital, the patient was conscious, but with difficulty speaking, grade 2 muscle strength of the left limb, grade 4 for the right limb, and positive Babinski’s sign on the left side. The neck rigidity was present and the Kernig sign was positive. Antifungal therapy started (voriconazole 200 mg bid, ivgtt). Dehydration (mannitol) to reduce intracranial pressure and low-dose glucocorticoids (methylprednisolone 40 mg qd, ivgtt) were also used. Two days later, the patient developed severe conjunctival congestion of the left eye with no light perception, and in less than one day, the left eye rapidly progressed to yellow-green vitreous opacity, with an unclear pupil (Fig. 4A).

Ophthalmic physical examination revealed no light perception in the left eye, hyperemia and Tyndall’s. The cornea was clear, however, a thick exudative membrane in the pupil was present, and the fundus was unclear. Eye ultrasound showed vitreous opacity in the left eye. The patient was treated with fluconazole and voriconazole eye drops combined with antibiotics. The infection was resolved, but her visual acuity was not improved. One week after the antifungal treatment, a head MRI (Fig. 3B) showed that the brain lesions became smaller in size, however severe edema around the lesions was still present. The lung CT (Fig. 1B) showed the lung lesions were significantly improved compared to the previous CT. The headache and dizziness were alleviated, and there was no significant change in muscle strength. Two weeks later, an orbital MRI (Fig. 4B and C) showed an abnormal signal of the left eyeball with enhancement. The patient’s muscle strength was significantly improved as the muscle strength of the left limb was grade 4-, and the right limb was grade 5. Two weeks later, the head MRI (Fig. 3C) and lung CT (Fig. 1C) showed that the abscess lesions became smaller. The pupil of the left eye was clear, but the eyeball atrophy and vision did not improve (Fig. 4D). After discharge, the patient was prescribed to continue voriconazole (200 mg bid po) and rehabilitation therapy.

**Fig. 5:**
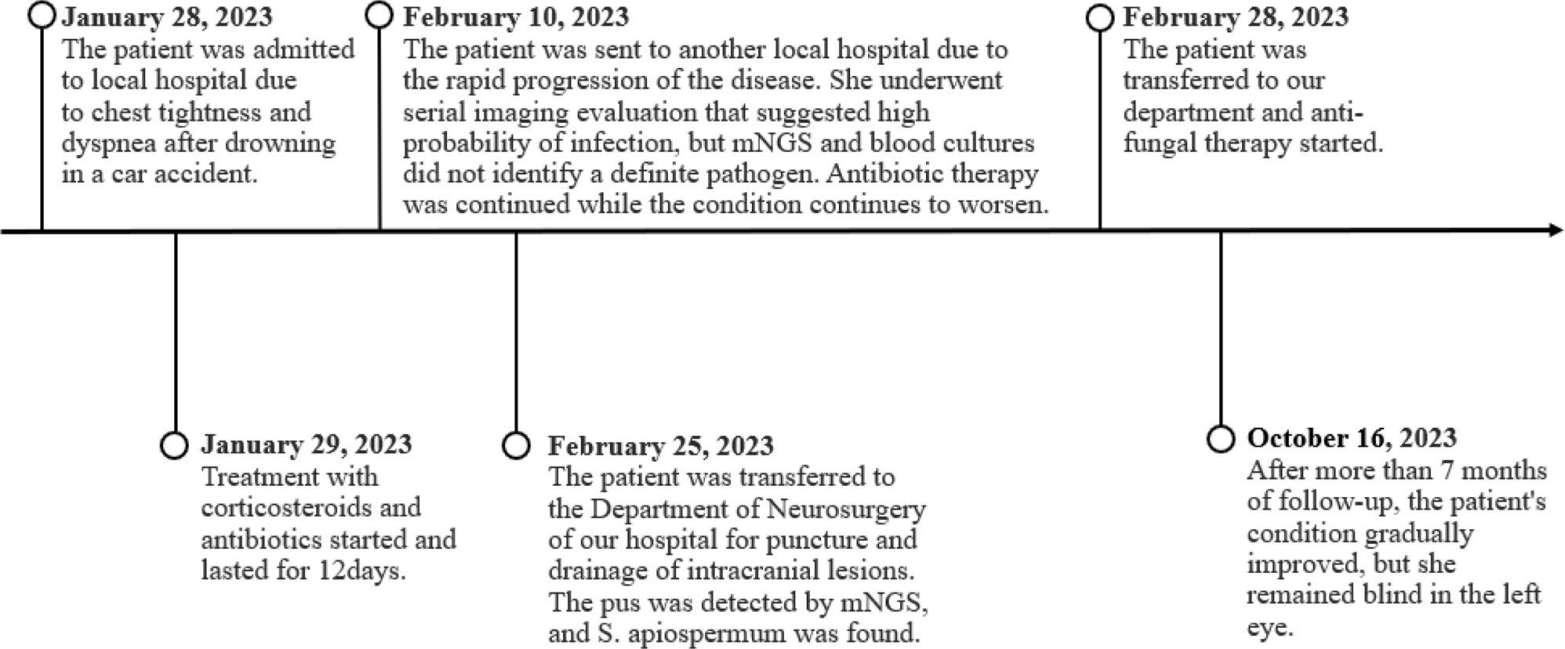
The timeline of the case

The patient has been followed up for over 7 months, and the brain and lung lesions were smaller than before, but have not completely disappeared (Figs. 1D and 3D). Figure 5 summarizes the timeline of the case.

## Discussion and conclusions

A study of 99 cases affecting the central nervous system from 1948 to 2007 found 44 cases of *S. apiospermum* infection that occurred in immunocompetent patients [3]. Among them, 55% had a history of drowning, 23% had post-traumatic/iatrogenic causes, and 10% had a history of alcohol abuse. Our case was an immunocompetent individual who was infected with *S. apiospermum* most probably after aspiration of contaminated water of fishpond. Severe and fast pulmonary, intracranial, and ocular symptoms, indicate that *S. apiospermum* has a potent toxicity and can invade the human body aggressively. Based on the case reports available in PubMed, we identified 13 cases of brain abscess limited to *S. apiospermum* infection after near drowning and finally analyzed the age and gender distribution, symptoms, treatment, and outcome of 14 cases after inclusion in our case ([see Additional file 1]). In these cases, the ratio of male to female was 11:3, and the mean age was 28 years. Other than the brain, the most common sites of infection were the lungs (57%), blood vessels (29%), and the eyes (29%). Fever was the most common symptom (71%), followed by respiratory distress (57%), poor consciousness (57%) and headache (50%). It is worth noting that other people within the same vehicle involved in this incident with near-drowning experience did not develop *S. apiospermum* infection.

This patient did not have a right-to-left shunt on echocardiography. It is inferred that this patient had obvious choking after near drowning compared with the other persons in the car and then hematogenic dissemination through the lungs that led to brain and eye infections [2]. Nuri Kiraz et al. [4] reported a case of lymphadenitis caused by *S. apiospermum* in an immunocompetent patient, who was initially presumed to be tuberculous lymphadenitis based on histopathological results and positive purified protein derivatives. After three months of anti-tuberculosis treatment, the number and size of lymph nodes continued to increase. Then, lymph node biopsy was repeated and *S. apiospermum* infection was confirmed. The treatment with oral itraconazole 200 mg tid reduced the diameter of lymph nodes within 6 months and the patient fully recovered within one year. In addition, Choon-Mee Kim et al. [5] reported a case of tenosynovitis, in which the patient was initially identified as having an Alternaria species infection by emulsion cotton blue staining microscopy. The *S. apiospermum* infection was confirmed through molecular DNA sequencing. 400 mg Fluconazole was intravenously infused once a day for 14 days and then changed to oral administration with half of the initial dosage. The whole course of treatment was 24 days. One week later, wound cultures for fungal growth were negative, and no recurrence reported during follow-up for four months. However, our case developed multiple lesions in multiple organs in a short period of time, which mimicked the metastatic tumor patterns on imaging, and had no fever and other signs of infection. Negative NGS and blood culture in the initial investigation resulted in the misdiagnosis of this case. We speculate that it may be attributed to the limitation of technical detection or the short onset time. The pathogen also needs a certain time to penetrate the blood-brain barrier (BBB) to replicate enough in blood or even cerebrospinal fluid. Therefore, mNGS was performed on the pus from the puncture and on blood culture again to identify the causative pathogen. According to the above cases, *S. apiospermum* infection can easily be misdiagnosed with tuberculosis and/or other fungal infections due to its low incidence and non-specific clinical manifestations. The combination of different diagnostic methods, such as histopathological examination, pathogenic bacteria culture, and imaging examination further improved the diagnosis rate. In those 14 cases we listed, more than one diagnostic method was used in half of the cases.

Moreover, for patients with primary negative results, it is necessary to closely monitor and test them multiple times to minimize the risk of infection remaining underdiagnosis particularly, as the disease starts to worsen and other possible causes have been ruled out. Moreover, obtaining samples from the lesion site has higher sensitivity and accuracy than the CSF. Unfortunately, due to patient refusal, we were unable to do a lumbar puncture or brain biopsy. However, the positive mNGS rate of up to 99.45% and the presence of *S. apiospermum* in blood culture were sufficient for our final diagnosis.

Due to its delayed diagnosis, severe complications, and lack of medication that can cross the BBB effectively, the *S. apiospermum* infections have a poor prognosis. Among these cases, seven cases were improved and seven cases died. Two of the deaths were not related to *S. apiospermum* infection itself, and the remaining deaths were due to complications associated with *S. apiospermum* infection. A fungal infection may have contributed to the cerebral hemorrhage that led to the death of one patient (Additional file 1; Case 3). Unlike bacterial aneurysms, fungal aneurysms are rare, often large, solitary, and ranging from 5 to 15 mm in diameter, usually involving the circle of Willis and the proximal arterial tree, readily enlarging and extending, and involving the length of the vessel wall which make endovascular or surgical treatment to be extremely difficult. mortality rate following an aneurysm rupture is close to 100% [6]. It is thought that fungal aneurysms occur due to direct vascular damage, either from fungal embolism of the vascular lumen or from the adventitia of the artery due to fungal meningitis [7]. Few cases with ocular involvement caused by *S. apiospermum* showed full recovery after prompt treatment [8, 9]. Ocular infection with *S. apiospermum* requires prompt local/topical antifungal therapy. Corneal transplantation or vitrectomy should be performed immediately if the response is not desirable. In our case, the causes of vision loss in the left eye were as follows: first, prompt antifungal therapy was not initiated as the diagnosis was unclear when mild vision loss and conjunctival congestion of the left eye occurred in the local hospital. Secondly, although local and systemic voriconazole treatment started after admission to our hospital, the intravitreal injection was not performed, hence the optimal intraocular penetration concentration failed to achieve, or the vitreous body was not removed in time led to the poor prognosis. In a review of 15 cases associated with *S. apiospermum* infection, treatments with amphotericin B and ketoconazole were associated with poor prognosis, whereas voriconazole and itraconazole were associated with better prognosis [10]. In the cases we listed, voriconazole was used in nine cases, including our case, which resulted in two deaths (22%) and an improvement of seven cases (78%). In particular, one patient (Additional file 1; case 5) showed no significant improvement with itraconazole use, but the symptoms were improved upon switching to voriconazole, providing more evidence about the effectiveness and efficacy of voriconazole. However, itraconazole, isoconazole, fluconazole, and miconazole shown to be not very effective (Additional file 1; case2, 3, 4, 5 and 12).

Following the combination of voriconazole, low-dose glucocorticoids, brain abscess drainage, surgery, and other medications, our patient had a relatively good prognosis. This might be due to the patient’s normal immune function and strong defense clearance ability. Voriconazole can effectively cross the BBB and reach the standard blood concentration, demonstrating the effectiveness of treatment. It’s reported that glucocorticoids are used in more than half of patients can reduce cerebral edema and brain abscesses [11]. Our case was treated with methylprednisolone 40 mg for severe cerebral edema. The headache was aggravated after glucocorticoid withdrawal. The headache was relieved upon glucocorticoid re-administration. One patient in our cases (Additional file 1; Case 13) also showed no significant aggravation after dexamethasone treatment. However, it has also been proposed that the use of glucocorticoids may lead to immunological dysregulation, which allows fungal invasion and dissemination [12, 13]. Therefore, the role of glucocorticoids in fungal treatment remains to be explored.

Moreover, even though the overall prognosis of the patient was good, the lesions in the brain and lung did not completely disappear during the follow-up of over five months. We later performed lymphocyte function testing, which did not reveal the presence of any latent immunodeficiency. In a review of 23 cases of scedosporiosis infection following near drowning, Aspasia Katragkou et al. [14] concluded that brain and lung lesions were resolved with long-term (> 1 year) voriconazole therapy. We therefore believe that long-term treatment is necessary.

*S. apiospermum* infection is rare, especially in immunocompetent individuals. Its diagnosis relies on pathogen culture and genome sequencing. However, in most cases, the prognosis of *S. apiospermum* infection is poor, and early medical treatment combined with surgical intervention may be effective. This report, emphasizes the importance of prompt detection of pathogens, which is associated with a higher survival rate and better prognosis. Meanwhile, fungal infection should be considered in rapidly progressing brain abscesses, and active pathogen identification should be performed.

### Patient perspective

The perspective of the patient was obtained during a follow-up of more than 7 months. The patient was generally satisfied with doctors’ treatment, but her only regret was the loss of vision in her left eye.

### Electronic supplementary material

Below is the link to the electronic supplementary material.

Supplementary Material 1

Supplementary Material 2

## Data Availability

The raw sequence data reported in this paper have been deposited in the Genome Sequence Archive in National Genomics Data Center, China National Center for Bioinformation / Beijing Institute of Genomics, Chinese Academy of Sciences with the accession number CRA015689.

## Abbreviations

*S. apiospermum*: *Scedosporium apiospermum*
mNGS: metagenomic next-generation sequencing
HIV: Human immunodeficiency virus
AIDs: Aquired immune deficiency syndrome
BBB: Blood brain barrier
