# Supplementary material for: Multi-organ involvement caused by Scedosporium apiospermum infection after near drowning: a case report and literature review
Source: BMC Neurol. 2024 Apr 15;24:124. doi: 10.1186/s12883-024-03637-9 (PMC11017585; doi:10.1186/s12883-024-03637-9)
Supplement: Supplementary file 1 — Supplementary Material 1 [file 12883_2024_3637_MOESM1_ESM.docx]

Table S1 Review of brain abscess due to S. apiospermum infection after near drowning

| **Case/**  **Refere**  **-nce** | **Age/**  **Sex** | | **Underlying conditions** | | **Symptoms** | | **Diagnosis** | | **Sites of**  **infection** | | **Treatment** | | **Outcome** | | **Year**  **Publis**  **-hed** | |
| --- | --- | --- | --- | --- | --- | --- | --- | --- | --- | --- | --- | --- | --- | --- | --- | --- |
| 1[15] | 32y/F | | Alcoholic cirrhosis,  polyneuritis | | Fever, respiratory failure, aspiration pneumonitis  coma, seizure, rash | | Culture(brain abscess), serology | | Brain, vessel | | 5- fluorocytosine,  amphotericin B,  Ketoconazole | | Death due to poor nutrition | | 1985 | |
| 2[16] | 2.5y/M | | None | | Fever, aspiration pneumonitis, somnolent, spastic paralysis | | Culture-histology (brain tissue) | | Brain,  eye, heart | | Dexamethasone, flucytosine, fluconazole | | Death due to central respiratory failure | | 1995 | |
| 3[17] | 3y/F | | None | | Fever, pulmonary edema, coma, hemiparesis, hydrocephalus | | Culture(brain abscess) | | Brain, vessel | | Surgery  + Itraconazole, | | Death | | 2002 | |
| 4[18] | 32y/M | | None | | Fever, cough, pulmonary infection, headache,  confusion, hydrocephalus, seizure, hemiparesis | | Culture (CSF) | | Brain, lung | | dexamethasone, amphotericin B,  itraconazole, cotrimoxazole,  fluconazole, miconazole | | Death | | 2004 | |
| 5[19] | 16m/M | | None | | Fever, ARDS, coma, spasticity, seizure, cortical blindness | | Fungal smear  (brain abscess) | | Brain, eye | | Surgery  + Itraconazole, voriconazole | | Recovered | | 2005 | |
| 6[20] | 39y/M | | None | | Fever, respiratory failure,  neuropsychiatric complications, renal failure, ventriculitis | | Culture(brain abscess),  PCR, histology | | Brain | | Surgery  + Voriconazole | | Death | | 2006 | |
| 7[21]  8[22] | 21y/M  51y/M | | None  None | | ARDS, headache, coma, brain herniation  Fever, headache, seizure, hydrocephalus, ventriculitis | | Culture-histology (brain tissue)  Culture (CSF) | | Brain,  kidney, vessel  Brain, vessel | | Surgery  + Caspofungin,  amphotericin B,  voriconazole  / | | Recovered  Death | | 2007  2010 | |
| **Case/**  **Refere**  **-nce** | | **Age/**  **Sex** | | **Underlying conditions** | | **Symptoms** | | **Diagnosis** | | **Sites of**  **infection** | | **Treatment** | | **Outcome** | | **Year**  **Publis**  **-hed** |
| 9[23] | | 36y/M | | None | | Fever, ARDS, cough, headache, pulmonary edema, pulmonary infection,  fungal endophthalmitis, vertebral osteomyelitis | | Culture(sputum, joint fluid) | | Brain,  lung, eye,  knee joint,  lumbar vertebrae | | Caspofungin, voriconazole, amphotericin B | | Recovered | | 2015 |
| 10[23] | | 29y/M | | None | | Fever, ARDS, pulmonary infection | | Culture  (sputum, bronchoalveolar lavage fluid) | | Brain, lung  eye,  thoracic vertebrae | | Voriconazole | | Recovered | | 2015 |
| 11[24] | | 62y/M | | None | | Fever, pulmonary infection,  headache, hydrocephalus | | Culture (CSF), PCR | | Brain, lung | | Voriconazole, terbinafine | | Recovered | | 2015 |
| 12[25] | | 13y/M | | None | | ARDS, aspiration pneumonia, headache,  poor consciousness, hydrocephalus, septic shock, renal failure, ventriculitis | | Culture  (brain abscess) | | Brain, lung | | Surgery + Amphotericin B, voriconazole, isoconazole | | Death | | 2018 |
| 13[26] | | 35y/M | | History  of substance  abuse | | Cough, aspiration pneumonia, limb weakness | | Culture(sputum), DNA  sequencing（sputum） | | Brain,  cervical cord | | Dexamethasone, voriconazole, terbinafine,  micafungin | | Recovered | | 2021 |
| 14(present case) | | 37y/F | | None | | Respiratory distress, pulmonary infection,  headache, Poor consciousness, limb weakness, poor vision | | Culture(blood), mNGS(brain abscess） | | Brain, lung,  Eye | | Surgery +  Methylprednisolone, voriconazole | | Recovered | | **/** |

*M*, Male. *F*, Female. *y*, year. *m*, month. *ARDS*, Acute Respiratory Distress Syndrome. *PCR*, polymerase chain reaction. *CSF*, cerebrospinal fluid.
